# Immunoproteasome Inhibition Modulates Microglial Polarization to Facilitate Anti-Inflammatory Responses and Hematoma Resolution After Intracerebral Hemorrhage

**DOI:** 10.3390/cells15080664

**Published:** 2026-04-09

**Authors:** Wei-Fen Hu, Chien-Hui Lee, Hsin-Yi Huang, Cheng-Yoong Pang, Yi-Feng Wu, Tsung-Jen Lin, Peter Bor-Chian Lin, Sheng-Tzung Tsai, Chia-Ho Lin, Hock-Kean Liew

**Affiliations:** 1 PhD Program in Pharmacology and Toxicology, College of Medicine, Tzu Chi University, Hualien 97004, Taiwan; 2 Department of Neurosurgery, Hualien Tzu Chi Hospital, Buddhist Tzu Chi Medical Foundation, Hualien 97002, Taiwan; 3 School of Medicine, Tzu Chi University, Hualien 97004, Taiwan; 4 Department of Medical Research, Hualien Tzu Chi Hospital, Buddhist Tzu Chi Medical Foundation, Hualien 97002, Taiwan; 5 Institute of Medical Sciences, Tzu Chi University, Hualien 97004, Taiwan; 6 Center for Cell Therapy and Development, Hualien Tzu Chi Hospital, Buddhist Tzu Chi Medical Foundation, Hualien 97002, Taiwan; 7 Department of Hematology and Oncology, Hualien Tzu Chi Hospital, Buddhist Tzu Chi Medical Foundation, Hualien 97002, Taiwan; 8 Division of Radiation Oncology, Department of Oncology, National Taiwan University Hospital, Taipei 10002, Taiwan; 9 Department of Neurology, Hope Center for Neurological Disorders, Knight Alzheimer’s Disease Research Center, Washington University in St. Louis, St. Louis, MO 63110, USA

**Keywords:** intracerebral hemorrhage (ICH), immunoproteasome, microglial polarization, phagocytosis, hematoma clearance

## Abstract

**Highlights:**

**What are the main findings?**

- Severe ICH activates microglial immunoproteasomes (LMP2/LMP7), accompanied by M1-like polarization, ER stress, and impaired hematoma clearance.
- Reduction in immunoproteasome expression attenuates maladaptive microglial responses and partially restores phagocytic function.

**What are the implications of the main findings?**

- Immunoproteasome expression may link hemorrhagic severity to dysfunctional microglial responses after ICH.
- Targeting immunoproteasome expression may help rebalance inflammatory and phagocytic activities after ICH.

**Abstract:**

Intracerebral hemorrhage induces severe secondary brain injury characterized by excessive neuroinflammation and inefficient hematoma clearance, processes largely governed by microglial polarization and phagocytic activity. The immunoproteasome, an inducible proteasome isoform involved in immune regulation, has been implicated in inflammatory neurological disorders, but its role in microglial responses after ICH remains unclear. In this study, rat models of common hemorrhage, severe hemorrhage, and severe hemorrhage with hematoma aspiration were used to represent graded injury severity and post-evacuation recovery. Transcriptomic profiling at day 3 post-injury identified immunoproteasome-associated gene networks, while expression of the catalytic subunits LMP2 and LMP7, microglial polarization markers, and phagocytic receptors was analyzed by Western blotting and immunofluorescence. Severe hemorrhage markedly induced LMP2 and LMP7 expression, predominantly in Iba1^+^ microglia, accompanied by enhanced ER stress, NF-κB signaling, and M1-like polarization and reduced phagocytic marker expression. Hematoma aspiration attenuated immunoproteasome expression and restored M2-associated and phagocytic signatures. Consistently, pharmacological inhibition of immunoproteasomes in primary microglia enhanced erythrophagocytosis and promoted a reparative phenotype in vitro. These findings indicate that immunoproteasome activation links hemorrhagic severity to maladaptive microglial polarization and impaired hematoma clearance after ICH, and that reducing immunoproteasome expression may help rebalance inflammatory and phagocytic microglial functions.

## 1. Introduction

Intracerebral hemorrhage (ICH) represents one of the most devastating subtypes of stroke, and is associated with exceptionally high rates of mortality and long-term disability [1,2]. The pathophysiology of ICH is characterized by a biphasic injury process. The primary injury arises from the rapid extravasation of blood into the brain parenchyma, resulting in mass effect, mechanical compression of neural tissue, and elevated intracranial pressure [3]. This is followed by a cascade of secondary injuries that evolve over hours to days, involving excitotoxicity, oxidative stress, and the toxic effects of hemoglobin, heme, and iron released from lysed erythrocytes [2,3,4]. Among these mechanisms, the inflammatory response mediated by resident immune cells (particularly microglia) plays a pivotal role in determining disease progression and recovery [5].

Microglia, the resident immune effector cells of the central nervous system, are rapidly activated in response to hematoma formation and cellular debris [6]. Their functional states are often described along an M1/M2 polarization spectrum [6,7]. M1-like microglia, characterized by markers such as CD86, CD14, and iNOS, secrete pro-inflammatory cytokines including TNF-α, IL-1β, and IL-6, thereby amplifying neuroinflammation [6,8]. In contrast, M2-like microglia, defined by the expression of Arginase-1 (Arg1), CD206 (mannose receptor), and Ym1 and the secretion of anti-inflammatory cytokines such as IL-10 and TGF-β1, contribute to erythrophagocytosis, hematoma clearance, and tissue repair [6,8]. The delicate balance between these opposing phenotypes has been implicated in various neurological disorders (e.g., Alzheimer’s disease, Parkinson’s disease, ischemic stroke), where an excessive M1 response and insufficient M2 activity contribute to neuronal degeneration and impaired recovery [6,8,9]. During the acute phase of ICH, the microglial response is predominantly skewed toward the M1 phenotype, while M2 polarization is transiently suppressed, leading to prolonged inflammation and delayed hematoma resolution [10,11].

Following ICH, activated microglia serve as the principal phagocytes responsible for engulfing extravasated red blood cells (RBCs), hemoglobin, and necrotic cellular debris from the hematoma [12,13]. Once internalized, these substrates are degraded through tightly coordinated intracellular clearance systems. The autophagy-lysosome pathway mediates the digestion of membrane-bound or particulate materials [14], while the ubiquitin-proteasome system (UPS) governs the selective degradation of oxidized or misfolded cytosolic proteins produced during oxidative and inflammatory stress [14,15]. Under conditions of massive hemolysis and iron accumulation, these degradation machineries become heavily burdened, resulting in protein aggregation and activation of cellular stress pathways [16]. Beyond simple waste disposal, the proteasome—particularly its inducible isoform, the immunoproteasome—plays a pivotal role in maintaining protein homeostasis and regulating immune signaling within activated microglia, thereby shaping the neuroinflammatory milieu after ICH [17,18,19].

Recent studies have highlighted the proteasome system as an important regulator of post-ICH neuroinflammation [20,21]. The proteasome, the major intracellular protein degradation machinery, exists in two main forms: the constitutive proteasome, expressed ubiquitously across tissues, and the immunoproteasome, which is preferentially induced under inflammatory and immune conditions [22,23]. The immunoproteasome comprises inducible catalytic subunits—LMP2 (β1i), LMP7 (β5i), and MECL-1 (β2i)—that not only facilitate antigen processing but also modulate inflammatory responses [24,25]. Upregulation of immunoproteasome activity has been observed in neurodegenerative diseases and after ischemic or hemorrhagic stroke, where it correlates with enhanced cytokine production and microglial activation [26,27,28]. Conversely, pharmacological inhibition of immunoproteasome activity, such as by ONX-0914, has been shown to suppress inflammatory cascades and mitigate tissue damage [29,30]. These findings suggest that the immunoproteasome may serve as a critical molecular hub linking hemorrhagic severity, microglial polarization, and secondary injury progression.

Although increasing evidence suggests that immunoproteasome activity is elevated under neuroinflammatory conditions, its specific role in shaping microglial responses after ICH has not been fully clarified. In particular, the mechanistic link between hemorrhagic injury, immunoproteasome regulation, and microglial functional states remains elusive. Therefore, this study was designed to investigate the relationship between ICH-induced immunoproteasome activation and its pharmacological inhibition on microglial polarization and phagocytic activity, aiming to determine whether modulation of immunoproteasome expression constitutes a critical regulatory pathway in post-hemorrhagic neuroinflammation and hematoma clearance.

## 2. Materials and Methods

### 2.1. Experimental Animals

All experimental procedures were approved by the Institutional Animal Care and Use Committee (IUCAC) of Tzu Chi Hospital, Taiwan (Approval Nos. IUCAC 108-60, 109-78 and 110-62), and were conducted in accordance with the National Institutes of Health Guide for the Care and Use of Laboratory Animals. Animals were maintained under a 12 h light/dark cycle with ad libitum access to food and water. Every effort was made to minimize animal suffering and to reduce the number of animals used.

A total of 57 adult male Sprague Dawley rats (300–350 g) were randomly assigned to two ICH severities. All surgical procedures were performed under isoflurane anesthesia (induction 3–5%, maintenance 2%). For terminal tissue collection, rats were deeply anesthetized with intraperitoneal pentobarbital sodium (50 mg/kg). Adequate depth of anesthesia was confirmed by the absence of a nociceptive withdrawal response to firm hind paw/toenail pinch (loss of pedal withdrawal reflex), after which animals were euthanized by decapitation or transcardially perfused, as appropriate for downstream analyses.

### 2.2. Induction of Intracerebral Hemorrhage and Hematoma Aspiration

#### 2.2.1. Common and Severe ICH Models

Collagenase was stereotaxically injected into the right striatum at coordinates 0.0 mm anterior, 3.0 mm lateral, and 5.0 mm ventral relative to bregma [31]. To establish the common ICH (I_C_) model, 0.2 U of collagenase VII-S dissolved in 1.0 μL sterile saline was administered, a protocol widely adopted for mild ICH [32,33]. In contrast, the severe ICH (I_S_) model was induced with 0.6 U collagenase VII-S in 3.0 μL saline, which consistently produced a larger hematoma and more severe neurological deficits [34].

#### 2.2.2. Hematoma Aspiration Procedure

For hematoma aspiration, severe ICH was first induced with 0.6 U collagenase. Six hours post-induction, when hematoma volume stabilized at approximately 200 mm^3^, rats were re-anesthetized, and a guide catheter was stereotaxically advanced through the hematoma cavity, from the ventral margin (0.0 mm anterior, 3.0 mm lateral, 7.5 mm ventral) to the dorsal margin (0.0 mm anterior, 3.0 mm lateral, 2.5 mm ventral). Hematoma was evacuated at a controlled withdrawal rate of 30 μL/min for 5 min, yielding a total aspiration volume of 150 μL. This procedure (I_S-HA_) removed approximately 75% of the total hematoma volume in the I_S_ model. The mortality rate in the severe hemorrhage (I_S_) model was approximately 30%, which is consistent with our previous report [35]. No unexpected mortality beyond that associated with injury severity was observed.

### 2.3. Lesion Volume Measurement

Brain lesion volumes were evaluated by morphometric analysis of coronal brain sections. At day 3 following ICH induction, rat brains were collected and sectioned coronally through the collagenase injection site, located approximately 0.00 mm relative to bregma, as previously described [36]. Serial 2-mm-thick slices were obtained both anterior and posterior to the injection site and photographed under standardized lighting conditions. Lesion areas in each section were delineated and quantified using ImageJ software 15.4 g (NIH, Bethesda, MD, USA). The total lesion volume (mm^3^) for each brain was then calculated and subjected to statistical analysis.

### 2.4. RNA Sequencing and Bioinformatic Analysis

Ipsilateral striatal tissue was collected at day 3 post-injury from I_C_, I_S_, and I_S-HA_ rats. Total RNA was extracted using the RNeasy Plus Mini Kit (Invitrogen, Carlsbad, CA, USA), and RNA integrity was confirmed using the Agilent 2100 Bioanalyzer (RIN ≥ 7.0). For library preparation, RNA was reverse-transcribed into complementary DNA (cDNA), and fragments of 250–300 bp were selectively enriched. The cDNA libraries were amplified by PCR and purified using AMPure XP magnetic beads (Beckman Coulter, Brea, CA, USA). Library quality and fragment size distribution were assessed using the Agilent 2100 Bioanalyzer, and library concentration was quantified by real-time PCR. Indexed libraries were sequenced on the Illumina NovaSeq 6000 platform (Illumina, San Diego, CA, USA) with a 150 bp paired-end format, generating a minimum of 30 million reads per sample to ensure adequate coverage.

Gene expression was quantified as transcripts per million (TPM), and differentially expressed genes (DEGs) were identified with DESeq2 using |log_2_ fold change| ≥ 1 and adjusted *p* < 0.05 as cutoffs. These thresholds were also applied for visualization in volcano plots and for subsequent downstream analyses. Functional enrichment analyses were performed for Gene Ontology (GO) and Kyoto Encyclopedia of Genes and Genomes (KEGG) pathways using the R package clusterProfiler. The cross-intersections of DEGs between I_S_ vs. I_C_ and I_S-HA_ vs. I_S_ were defined as genes that were upregulated in I_S_ vs. I_C_ and downregulated in I_S-HA_ vs. I_S_, and were identified and visualized using Venn diagram analysis. The overlapping DEGs were further subjected to upstream regulator prediction and canonical pathway analysis using Ingenuity Pathway Analysis (IPA, Qiagen, Hilden, Germany). Protein-protein interaction networks were generated with STRING and visualized in Cytoscape 3.10.4.

### 2.5. Tissue Protein Extraction

Ipsilateral and contralateral striatum tissue were lysed with T-per (Thermo Fisher, Waltham, MA, USA) containing 1% protease & phosphatase inhibitor single-use cocktail, EDTA-free (Thermo Fisher, Waltham, MA, USA), grinded on ice, then centrifuged at 13,200 rpm at 4 °C for 20 min. After centrifugation, the supernatant was taken as the tissue protein solution.

### 2.6. Western Blot

Sample solutions containing 40 μg of protein were separated on 10% SDS-polyacrylamide gels and transferred to immobilon polyvinylidene difluoride membranes (PVDF; Millipore). To block the nonspecific binding, the membranes were incubated with 5% skim milk in TBST buffer (0.1 M Tris-HCl (pH 7.4), 0.9% NaCl, 0.1% Tween 20) for 1 h at room temperature. After rinsing with TBST buffer, membranes were incubated with specific primary antibodies (LMP2, 1:1000, MYBioSource, San Diego, CA, USA; LMP7, 1:1000, MYBioSource; GRP78, 1:1000, Cell Signaling, Danvers, MA, USA; CHOP, 1:1000, Cell signaling; p-NF-κB, 1:1000, Cell signaling; MPO, 1:5000, abcam, Cambridge, UK; CD14, 1:1000, ABclonal, Wuhan, China; Arginase-1, 1:10,000, ABclonal; CD163, 1:1000, Bioss, Beijing, China; CD36, 1:1000, ABclonal; CD68, 1:1000, Bio-Rad, Hercules, CA, USA; actin, 1:20,000, Sigma, St. Louis, MO, USA) overnight in 4 °C. Then, membranes were incubated with appropriate HRP-conjugated secondary antibodies, and the proteins were detected using the Western Lightning Plus-ECL (PerkinElmer, Waltham, MA, USA). The chemiluminescence was visualized using the iBright FL1500 Imaging System (Thermo Fisher Scientific).

### 2.7. Immunofluorescence Staining

After deep anesthesia, rats were perfused with saline and 4% paraformaldehyde through the left ventricle. Brains were translocated and post-fixed in 4% paraformaldehyde at room temperature for 2 h, cryoprotected in 30% (*w*/*v*) sucrose (4 °C), embedded in Tissue-Tek O.C.T. compound, frozen, and stored at −80 °C until further analysis. Serial (20 μm) coronal sections were sliced on a freezing slicer. Tissue sections were blocked with 2% fetal bovine serum in PBS, then incubated overnight with primary antibodies (Iba1, 1:100, Wako, Osaka, Japan; LMP2, 1:100, MYBioSource; LMP7, 1:100, MYBioSource; CD86, 1:100, ABclonal; Arginase-1, 1:200, ABclonal; CD163, 1:100, Bioss; NeuN, 1:1000, abcam; GFAP, 1:200, GeneTex, Irvine, CA, USA; RECA, 1:200, abcam). After washing, secondary antibodies, which were conjugated to the fluorescent markers Alexa Fluor™ Plus 488/647/555 (Thermo Fisher, Waltham, MA, USA), were applied to sections for 1 h. Sections were then washed, mounted on slides, cover-slipped with Vectashield mounting medium (Thermo Fisher, Waltham, MA, USA), and examined with a fluorescence microscope (Carl Zeiss, Oberkochen, Germany).

### 2.8. Microglia Cell Culture

As previously described, primary microglial cells were isolated from postnatal day 1–2 (P1–P2) rat pups [31]. Briefly, neonatal pups were euthanized, and the ventral mesencephalic tissue was isolated through mild mechanical trituration. The isolated cells (5 × 10^7^) were plated in 150 cm^2^ culture flasks containing DMEM supplemented with 10% FBS, 50 U/mL penicillin, and 50 mg/mL streptomycin and were cultured for 14 days. Loosely adherent microglia were collected by shaking the flasks at 180 rpm for 2 h at 37 °C. A total of 2 × 10^5^ cells per well were plated in 24-well plates and incubated for 24 h before undergoing drug treatments or phagocytosis assays.

### 2.9. In Vitro Erythrophagocytosis Assay

The red blood cells (RBCs) were isolated from the whole blood of donor rats using Ficoll-Paque PLUS (GE Healthcare, Chicago, IL, USA). The purified RBCs were then labeled with the fluorescent dye 5(6)-carboxyfluorescein diacetate (CFDA; Molecular Probes, Eugene, OR, USA) for use in in vitro studies. Primary microglia were co-cultured with CFDA-SE-labeled RBCs at a 1:10 ratio in a 37 °C incubator. After 3 h, the cultures were washed three times with Dulbecco’s PBS to remove any non-engulfed RBCs. Microglial cells were lysed with sterile water to facilitate the release of fluorescent material. The phagocytic index was then measured using a spectrophotometer, or alternatively, microglial cells were fixed with 4% paraformaldehyde (PFA) for 10 min and stained with Iba1. Engulfed RBCs were visualized under a fluorescent microscope.

### 2.10. Statistical Analysis

Data are presented as mean  ±  SEM. Statistical analysis was performed using Prism software (GraphPad Software 11, San Diego, CA, USA). Figures were obtained using Student’s *t*-test. The mNSS scores were statistically analyzed using one-way ANOVA. A *p*-value less than 0.05 is considered statistically significant.

## 3. Results

### 3.1. Transcriptomic Analysis Reveals Immunoproteasome-Associated Gene Regulation Following Hemorrhagic Injury

**Figure 1:**
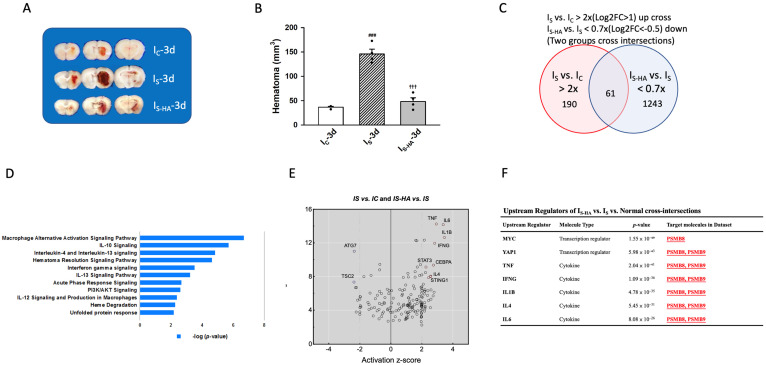
Transcriptomic analysis reveals immunoproteasome-associated gene regulation after hemorrhagic injury. (**A**) Representative coronal sections at day 3 showing moderate hematoma formation in I_C_, larger hematoma volume in I_S_, and marked reduction following hematoma aspiration in I_S-HA_. (**B**) Quantification of hematoma volume demonstrates significant increases in I_S_ and attenuation in I_S-HA_ (*n* = 4 animals per group). (**C**) Venn diagram of differentially expressed genes showing 190 genes upregulated in I_S_ versus I_C_ and 1243 genes downregulated in I_S-HA_ versus I_S_, with 61 genes overlapping. Differentially expressed genes were defined using thresholds of >2-fold increase (log2FC > 1) for I_S_ vs. I_C_ and <0.7-fold decrease (log2FC < −0.5) for I_S-HA_ vs. I_S_. The overlapping genes represent the intersection of the two comparisons (I_S_ vs. I_C_ and I_S-HA_ vs. I_S_). (**D**) Pathway enrichment analysis of the intersecting gene set indicates enrichment of immune- and inflammation-related signaling pathways, including IL-10 signaling, IL-4/IL-13 signaling, hematoma resolution signaling, IFN-γ signaling, PI3K/AKT signaling, and the unfolded protein response. (**E**) Upstream regulator activation analysis of the intersecting gene set using Ingenuity Pathway Analysis (IPA). Each dot represents a predicted upstream regulator, plotted according to activation z-score (*x*-axis) and statistical significance (-log *p*-value, *y*-axis). Selected regulators associated with inflammatory signaling are highlighted. (**F**) Representative upstream regulators predicted from the intersecting gene set, including inflammatory cytokines (TNF, IFN-γ, IL-1β, IL-4, IL-6) and transcriptional regulators (MYC, YAP1). Within this predicted regulatory network, the immunoproteasome genes Psmb8 (LMP7) and Psmb9 (LMP2) were identified as shared downstream targets of multiple regulators. RNA-seq analyses were performed with *n* = 3 biological replicates per group. Data are expressed as mean ± SEM. ### *p* < 0.001 versus I_C_; ††† *p* < 0.001 versus I_S_.

To elucidate the molecular responses underlying different severities of hemorrhagic injury and the effect of hematoma evacuation, three rat models were established: common hemorrhage (I_C_), severe hemorrhage (I_S_), and severe hemorrhage with hematoma aspiration (I_S-HA_). Representative coronal sections at day 3 post-injury demonstrated distinct hematoma characteristics among the three experimental conditions. In the I_C_ group, moderate hematoma formation was observed, whereas the I_S_ group exhibited substantially larger hematoma volumes. In contrast, hematoma aspiration markedly reduced lesion volume in the I_S-HA_ group (Figure 1A,B). Transcriptomic profiling revealed substantial alterations in gene expression across these conditions. A total of 190 genes were upregulated in I_S_ compared with I_C_, whereas 1243 genes were downregulated in I_S-HA_ relative to I_S_, with 61 genes overlapping between the two comparisons (Figure 1C). Importantly, although Psmb8 and Psmb9 were not included in this overlapping gene set, both genes exhibited consistent regulation across conditions and were identified as shared downstream targets of multiple predicted upstream regulators (Figure 1F), highlighting their biological relevance. Pathway enrichment analysis showed that these intersecting genes were significantly associated with immune- and inflammation-related signaling pathways, including macrophage alternative activation, IL-10 signaling, IL-4/IL-13 signaling, hematoma resolution signaling, interferon-γ signaling, acute phase response, PI3K/AKT signaling, heme degradation, and the unfolded protein response (Figure 1D). Upstream regulator analysis further identified transcription factors and cytokines—including MYC, YAP1, TNF, IFN-γ, IL-1β, IL-4, and IL-6—as major drivers of these transcriptomic changes (Figure 1E,F). Notably, key inflammatory regulators such as TNF, IL-1β, IFN-γ, IL-4, and IL-6 converged on the immunoproteasome catalytic subunits PSMB8 (LMP7) and PSMB9 (LMP2), highlighting a central role of the immunoproteasome in cytokine-driven inflammatory signaling following ICH. These findings suggest that hematoma severity modulates inflammatory activation through immunoproteasome-associated pathways, whereas hematoma aspiration attenuates excessive pro-inflammatory responses. Based on this, day 3 post-injury was selected for analysis as a representative phase of peak inflammation [37,38]. Consistently, our time-course data showed marked upregulation of LMP2 and LMP7, with CD68 expression also peaking at this time point (Supplementary Figure S1). Furthermore, correlation analysis across hemorrhagic models (Supplementary Figure S3) revealed that hematoma volume was positively associated with LMP2 and LMP7 expression, indicating that increased hemorrhagic burden is linked to enhanced immunoproteasome activation.

### 3.2. Severe Hemorrhage Induces Immunoproteasome Activation, Attenuated by Hematoma Aspiration

**Figure 2:**
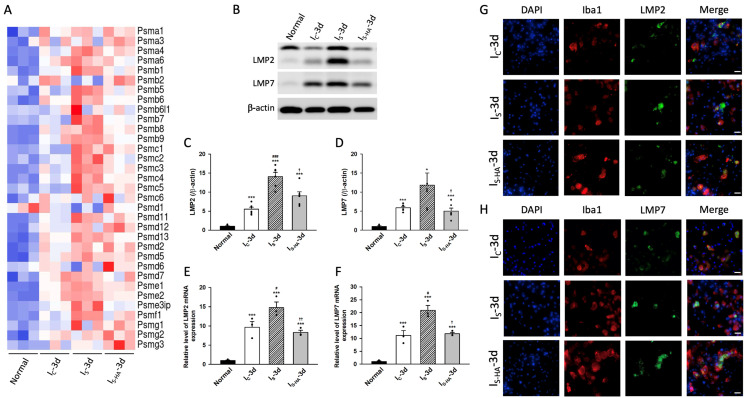
Severe hemorrhage induces immunoproteasome activation, attenuated by hematoma aspiration. (**A**) Heatmap of proteasome gene expression showing increased Psmb8 (LMP7) and Psmb9 (LMP2) in I_S_ and reduced expression in I_S-HA_ (*n* = 3 biological replicates per group). (**B**) Representative Western blots of LMP2 and LMP7 proteins. (**C**,**D**) Quantification showing significant upregulation of LMP2 and LMP7 in I_S_ and reduction in I_S-HA_ (*n* = 6 animals per group). (**E**,**F**) RNA sequencing analysis showing relative expression changes of LMP2 and LMP7 transcripts across groups, consistent with protein expression patterns (*n* = 3 biological replicates per group). (**G**,**H**) Immunofluorescence staining showing colocalization of LMP2 or LMP7 (green) with Iba1^+^ microglia (red), strongest in I_S_ and attenuated in I_S-HA_. Scale bars = 20 μm. Data are expressed as mean ± SEM. * *p* < 0.05, *** *p* < 0.001 versus Normal; # *p* < 0.05, ### *p* < 0.001 versus I_C_; † *p* < 0.05, †† *p* < 0.01 versus I_S_.

To corroborate the transcriptomic findings, we next examined the expression of immunoproteasome subunits at both the transcript and protein levels. Heatmap visualization revealed differential regulation of proteasome-related genes across experimental groups, with Psmb8 and Psmb9 showing the most consistent and pronounced upregulation in I_S_ compared with I_C_, and partial reduction in I_S-HA_ (Figure 2A). Consistent with these findings, Western blot analysis demonstrated that both LMP2 and LMP7 protein levels were markedly increased in I_S_, moderately elevated in I_C_, and significantly reduced in I_S-HA_ (Figure 2B–D). Densitometric quantification normalized to β-actin confirmed these differences. Notably, two bands were observed for LMP2, which likely represent precursor and processed forms of the protein, consistent with previous reports of immunoproteasome maturation. Transcription analysis further confirmed parallel transcriptional upregulation of LMP2 and LMP7 in I_S_, with partial normalization following hematoma aspiration (Figure 2E,F). Together, these results demonstrate that immunoproteasome activation is positively associated with hemorrhage severity and is attenuated following hematoma removal. To determine the cellular localization of immunoproteasome induction, double immunofluorescence staining was performed. LMP2^+^ and LMP7^+^ signals were predominantly associated with Iba1^+^ microglia in the perihematomal region (Figure 2G,H). However, to address the possibility that these signals might originate from engulfed dying cells rather than the microglia themselves, additional confocal z-stack imaging with optical sectioning and orthogonal reconstruction was performed (Supplementary Figure S2). These analyses demonstrated that LMP7 signals were localized within Iba1^+^ microglial cell bodies in three-dimensional space, supporting true intracellular expression rather than extracellular debris. Furthermore, minimal colocalization of LMP7 with NeuN^+^ neuronal cell bodies was observed, suggesting that apparent neuronal signals in conventional imaging may reflect phagocytosed material rather than intrinsic neuronal expression. Single-cell analysis further confirmed that LMP7-positive signals were confined within Iba1-positive microglia. Collectively, these findings provide strong evidence that immunoproteasome activation following hemorrhagic injury occurs predominantly within microglia and correlates with hemorrhage severity.

### 3.3. Immunoproteasome Expression Is Positively Associated with ER Stress, Inflammatory Signaling, and Microglial Polarization After Hemorrhagic Injury

**Figure 3:**
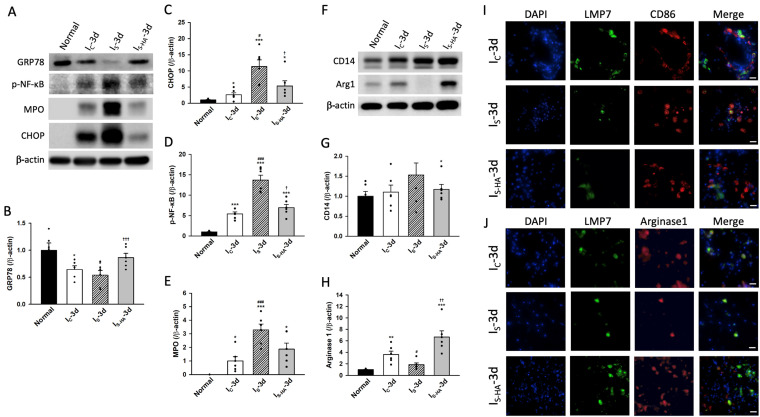
Immunoproteasome expression is positively associated with ER stress, inflammatory signaling, and microglial polarization after hemorrhagic injury. (**A**) Representative Western blot images of ER stress and inflammatory markers showing increased CHOP, p-NF-κB, and MPO expression in I_S_, with a modest overall change in GRP78; hematoma aspiration partially reduced CHOP, p-NF-κB, and MPO levels. (**B**,**C**) Quantification of GRP78 (**B**) and CHOP (**C**) protein levels normalized to β-actin (*n* = 6 animals per group). (**D**,**E**) Quantification of p-NF-κB (**D**) and MPO (**E**) protein levels normalized to β-actin (*n* = 6 animals per group). (**F**) Representative Western blot images of microglial-associated markers showing a clear trend toward increased CD14 expression and reduced Arg1 expression in I_S_; these changes were partially reversed in I_S-HA_. (**G**,**H**) Quantification of CD14 (**G**) and Arg1 (**H**) protein levels normalized to β-actin (*n* = 6 animals per group). (**I**,**J**) Representative immunofluorescence images showing colocalization of LMP7 (green) with CD86 (red, **I**) or Arg1 (red, **J**). LMP7-CD86 colocalization was more prominent in I_S_-3d, whereas LMP7-Arg1 colocalization was markedly increased in I_S-HA_-3d. Quantitative analysis of CD86^+^ or Arg1^+^ cells co-expressing LMP7 is presented in Supplementary Figure S5. Nuclei were stained with DAPI (blue). Scale bars = 20 μm. Data are expressed as mean ± SEM. * *p* < 0.05, ** *p* < 0.01, *** *p* < 0.001 versus Normal; # *p* < 0.05, ### *p* < 0.001 versus I_C_; † *p* < 0.05, †† *p* < 0.01, ††† *p* < 0.001 versus I_S_.

To determine how immunoproteasome activation relates to inflammatory signaling and microglial phenotype, we examined ER stress markers, inflammatory mediators, and polarization-associated proteins. Western blot analysis showed that CHOP was markedly elevated in I_S_, whereas GRP78 exhibited only a modest increase compared with I_C_ and did not reach statistical significance across groups. CHOP levels were partially reduced following hematoma aspiration (Figure 3A–C). These findings suggest activation of ER stress signaling, with CHOP representing the more robustly regulated component. Consistent with these findings, a similar pattern of ER stress regulation was observed across different hemorrhagic models (Supplementary Figure S4), further supporting a severity-dependent activation of ER stress pathways. Inflammatory activation was also enhanced in I_S_, as indicated by increased phosphorylated NF-κB and MPO levels, both of which were attenuated following hematoma aspiration (Figure 3A,D,E). However, these signals likely reflect contributions from multiple cell types within the injured brain tissue rather than being restricted to microglia. Analysis of microglial polarization markers revealed that the M1-associated protein CD14 showed a trend toward an increase in I_S_ without reaching statistical significance, whereas the M2 marker Arg1 was reduced. Hematoma aspiration partially reversed these changes, with increased Arg1 expression and a tendency toward reduced CD14 levels (Figure 3F–H). To further assess microglial polarization at the cellular level, triple immunofluorescence staining and quantitative analysis were performed. LMP7 showed prominent colocalization with the M1 marker CD86 in I_S_ (Figure 3I). Quantitative analysis demonstrated that the proportion of Iba1^+^CD86^+^LMP7^+^ cells relative to total Iba1^+^LMP7^+^ microglia was significantly increased in I_S_ and reduced in I_S-HA_ (Supplementary Figure S5A). Conversely, LMP7 exhibited increased colocalization with the M2 marker Arg1 in I_S-HA_ (Figure 3J). Quantitative analysis showed that the proportion of Iba1^+^Arg1^+^LMP7^+^ cells was significantly elevated in I_S-HA_ compared with I_S_ (Supplementary Figure S5B). These findings indicate a shift from a pro-inflammatory phenotype in I_S_ toward a more anti-inflammatory and phagocytic phenotype following hematoma aspiration. Overall, immunoproteasome activation is associated with ER stress and inflammatory signaling and is preferentially linked to M1-like microglial features under severe hemorrhagic conditions, whereas attenuation of this activation is accompanied by a transition toward a more reparative phenotype.

### 3.4. Immunoproteasome Colocalization with Phagocytic Markers in Microglia

**Figure 4:**
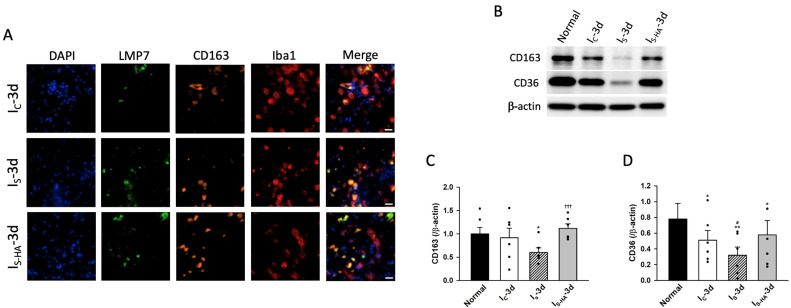
Immunoproteasome colocalization with phagocytic markers in microglia. (**A**) Immunofluorescence staining showing colocalization of LMP7 (green) with CD163 (orange) in Iba1^+^ microglia (red) across I_C_, I_S_, and I_S-HA_ groups. Colocalization intensity was reduced in I_S_, whereas the strongest colocalization was observed in I_S-HA_. (**B**) Representative Western blots of CD163 and CD36 expression. (**C**,**D**) Quantification of CD163 and CD36 protein expression normalized to β-actin (*n* = 6 animals per group). Severe hemorrhage reduced CD163 and CD36 levels, both of which were markedly restored after hematoma aspiration. Data are expressed as mean ± SEM. (*n* = 5 per group). * *p* < 0.05, ** *p* < 0.01 versus Normal; # *p* < 0.05 versus I_C_; ††† *p* < 0.001 versus I_S_.

Given that erythrocyte lysis after ICH releases hemoglobin and other toxic byproducts that exacerbate secondary injury, efficient clearance of hematoma-derived debris by microglia is essential for neuroprotection [12,39,40,41,42]. To investigate whether immunoproteasome activation is associated with phagocytic responses, we examined the colocalization of LMP7 with phagocytosis-related markers. Immunofluorescence staining revealed that LMP7 was colocalized with CD163^+^ and Iba1^+^ microglia in the perihematomal region (Figure 4A). Quantitative analysis demonstrated that the proportion of Iba1^+^CD163^+^LMP7^+^ cells was significantly reduced in I_S_ compared with I_C_, and significantly increased in I_S-HA_ relative to I_S_ (Supplementary Figure S6A). Western blot analysis further showed that CD163 and CD36 protein levels were both reduced in I_S_ and partially restored following hematoma aspiration (Figure 4B–D). Although CD163 is commonly associated with M2-like microglia and CD36 with phagocytic function [43,44,45], their simultaneous reduction in I_S_ suggests a globally impaired microglial state under severe hemorrhagic stress rather than a simple M1/M2 shift. This interpretation is supported by the concurrent increase in inflammatory and ER stress markers. Importantly, the restoration of CD163 and CD36 expression in I_S-HA_ indicates recovery of microglial phagocytic capacity following reduction in hemorrhagic burden.

### 3.5. Immunoproteasome Inhibition Enhances Microglial Phagocytosis In Vitro

**Figure 5:**
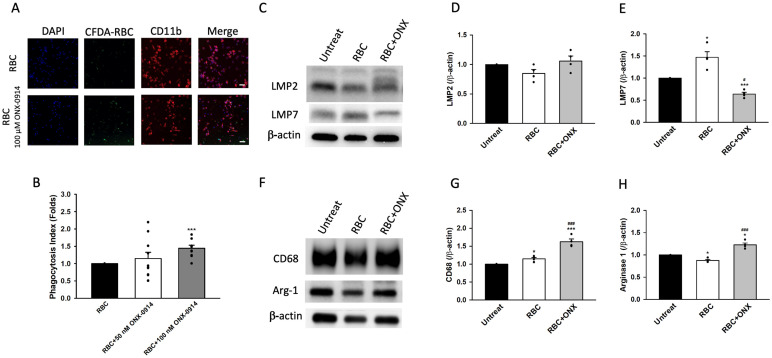
Immunoproteasome inhibition enhances microglial phagocytosis in vitro. (**A**) Immunofluorescence images showing CFDA-labeled RBCs (green) incubated with primary microglia, with CD11b marking microglia (red). ONX-0914 (100 nM) increased RBC uptake, as indicated by enhanced CFDA-RBC signals within CD11b^+^ cells. (**B**) Quantification of phagocytosis index demonstrating a dose-dependent increase in erythrophagocytosis, with 100 nM ONX-0914 producing the greatest enhancement (*n* = 10 wells per group). (**C**) Western blot analysis showing that RBC stimulation upregulated LMP7 expression, whereas ONX-0914 treatment reduced LMP7 levels; LMP2 showed no significant changes. (**D**,**E**) Quantification of LMP2 and LMP7 protein levels normalized to β-actin (*n* = 4 biological replicates per group). (**F**) Western blots showing expression of phagocytic (CD68) and M2-associated (Arg1) markers in untreated, RBC-treated, and RBC + ONX-0914 microglia. (**G**,**H**) Quantification showing that RBC exposure increased CD68 levels, and ONX-0914 further elevated both CD68 and Arg1 expression, indicating enhanced phagocytic activation and partial M2 polarization (*n* = 4 biological replicates per group). Data are presented as mean ± SEM. * *p* < 0.05, *** *p* < 0.001, versus Untreat; # *p* < 0.05, ### *p* < 0.001 versus RBC.

To directly assess the functional role of immunoproteasomes in microglial phagocytosis, we established an in vitro erythrophagocytosis assay using primary microglia cocultured with CFDA-labeled RBCs in the presence or absence of the selective immunoproteasome inhibitor ONX-0914. Fluorescence imaging and quantitative analysis demonstrated that ONX-0914 significantly enhanced microglial erythrophagocytosis in a dose-dependent manner, with 100 nM producing a marked increase in the phagocytosis index (Figure 5A,B). At the molecular level, RBC exposure increased LMP7 expression, whereas ONX-0914 treatment effectively reduced LMP7 protein levels without significantly altering LMP2 expression (Figure 5C–E), indicating selective inhibition. RBC stimulation increased CD68 expression, and ONX-0914 further enhanced CD68 levels (Figure 5F,G). In addition, Arg1 expression was reduced following RBC exposure but restored and significantly increased with ONX-0914 treatment (Figure 5F–H). These results demonstrate that pharmacological inhibition of the immunoproteasome enhances microglial phagocytosis while promoting a shift toward a reparative, M2-like phenotype. Together with the in vivo findings, these data support a functional role of immunoproteasomes in regulating microglial phenotype and phagocytic capacity following intracerebral hemorrhage.

Following ICH, hemolysis-derived hemoglobin, heme, and iron trigger microglial activation and markedly induce immunoproteasome signaling. Excessive immunoproteasome activity promotes classical activation of microglia toward a proinflammatory M1 phenotype, enhancing inflammatory responses and contributing to impaired hematoma resolution. In contrast, inhibition of immunoproteasome activity by ONX-0914 suppresses M1-associated inflammatory signaling and facilitates alternative activation toward an M2 anti-inflammatory phenotype. This shift enhances microglial erythrophagocytosis and promotes more efficient hematoma clearance after ICH.

## 4. Discussion

In this study, we identify immunoproteasome activation as a central mechanism linking hemorrhagic burden to neuroinflammation and impaired microglial clearance after ICH. Transcriptomic and proteomic analyses consistently demonstrated robust induction of the immunoproteasome subunits LMP2 and LMP7 in severe hemorrhage, with a clear temporal increase observed during the early and subacute phases after ICH (Supplementary Figure S1), accompanied by increased TNF-α, IL-1β, and phosphorylated NF-κB, together with suppression of phagocytosis-related markers including CD163 and CD36. These findings indicate that excessive immunoproteasome activation is closely associated with pro-inflammatory microglial polarization and reduced erythrophagocytic capacity. Hematoma aspiration markedly attenuated LMP2/LMP7 expression, reduced inflammatory signaling, and restored phagocytic marker expression, suggesting that removal of blood-derived components alleviates immunoproteasome-associated neuroinflammation. Consistently, pharmacological inhibition with ONX-0914 enhanced erythrophagocytosis and promoted anti-inflammatory polarization in cultured microglia. Collectively, these data support the immunoproteasome as a key regulator of microglial activation and clearance functions after ICH.

Importantly, the present study focused on day 3 post-ICH as the primary time point for analysis. This selection was based on both our preliminary observations and previous literature indicating that day 3 represents a critical phase of peak inflammatory activation and predominant M1-like microglial polarization after ICH. Consistent with this, our time-course analysis demonstrated robust upregulation of immunoproteasome subunits LMP2 and LMP7 at this time point (Supplementary Figure S1). In addition, phagocytic markers such as CD68 have been reported to peak around day 3, reflecting an active microglial response during the early subacute phase. Our own data similarly showed maximal CD68 expression at day 3 following ICH. Therefore, this time point was selected to capture the most pronounced immunoproteasome-associated inflammatory and microglial responses.

Building upon our previous work demonstrating that hematoma evacuation mitigates mitochondrial dysfunction and secondary injury, the present findings further clarify the upstream drivers of immunoproteasome activation. Comparative analysis across hemorrhagic models revealed that blood infiltration, rather than mechanical compression alone, is a primary stimulus for immunoproteasome induction. The balloon compression model showed minimal LMP2/LMP7 upregulation, whereas autologous blood injection markedly increased immunoproteasome expression. Moreover, hematoma volume positively correlated with LMP7 protein levels (Supplementary Figure S3), further supporting a hemorrhage-dependent regulation of immunoproteasome activation. Together, these findings support a model in which hemorrhagic burden amplifies inflammatory proteolytic activity in perihematomal tissue. Mechanistically, immunoproteasome activation appears to coordinate both inflammatory signaling and microglial functional responses. The temporal association between LMP7 expression and CD68 suggests a potential link between immunoproteasome activity and phagocytic processes during the subacute phase of injury. Although immunoproteasome induction was observed in multiple cell types, microglia exhibited predominant colocalization with LMP7, supporting their major contribution to inflammatory and clearance responses after ICH.

ER stress represents an important downstream pathway associated with immunoproteasome activation. In the severe ICH model, GRP78 showed only modest changes whereas CHOP was markedly upregulated, accompanied by increased p-NF-κB and MPO expression (Supplementary Figure S4), indicating aggravated proteostatic stress and inflammatory activation. Hematoma aspiration partially restored ER stress markers and attenuated LMP2/LMP7 expression, suggesting that immunoproteasome activity is dynamically influenced by hematoma-derived oxidative and proteotoxic stress. These findings support a model in which excessive hemorrhagic burden enhances immunoproteasome activation and ER stress, forming a maladaptive inflammatory loop that may exacerbate secondary neuronal injury.

While ONX-0914 effectively enhanced erythrophagocytosis and anti-inflammatory polarization in vitro, translation to in vivo settings requires further optimization. Factors such as drug delivery, bioavailability, and tissue penetration may influence therapeutic efficacy under severe hemorrhagic conditions. Future studies employing optimized pharmacological strategies or genetic approaches will be necessary to fully evaluate the therapeutic potential of immunoproteasome inhibition in ICH.

Several limitations should be acknowledged. First, residual tissue hemoglobin was not directly quantified, and hematoma volume was used as a surrogate marker of hemorrhagic burden. Second, although microglia exhibited predominant colocalization with LMP7, contributions from other neural cell types cannot be entirely excluded. Third, while strong associations were observed, further studies are needed to delineate the specific molecular pathways linking blood-derived components to immunoproteasome activation. Fourth, although pharmacological inhibition of the immunoproteasome showed beneficial effects on microglial polarization and erythrophagocytosis in vitro, translation to in vivo settings requires further optimization, including dosing strategies, delivery routes, and tissue penetration. Future investigations using cell-specific and mechanistic approaches will help refine these regulatory pathways and clarify the therapeutic potential of immunoproteasome targeting in ICH.

Overall, our findings establish the immunoproteasome as a central molecular regulator linking hemorrhagic burden to inflammatory amplification and impaired microglial clearance after ICH. By integrating hematoma volume, ER stress, and microglial polarization into a unified framework, this study advances our understanding of secondary injury mechanisms and highlights immunoproteasome modulation as a potential therapeutic target.

**Figure 6:**
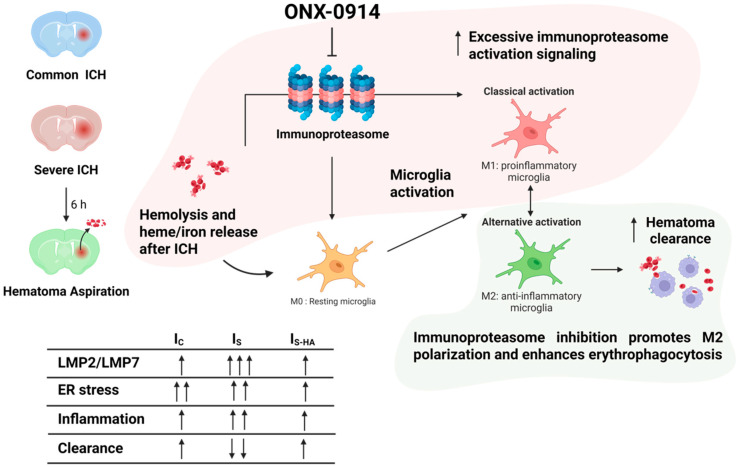
Schema of immunoproteasome inhibition-enhanced hematoma resolution.

As summarized in Figure 6, hemorrhage-induced hemolysis drives immunoproteasome activation, promoting pro-inflammatory microglial polarization and impaired hematoma clearance, whereas immunoproteasome inhibition shifts microglia toward a reparative phenotype that enhances erythrophagocytosis and resolution.

## 5. Conclusions

This study identifies the immunoproteasome as a key mediator linking inflammation, ER stress, and impaired microglial clearance after ICH. Excessive LMP2/LMP7 activation drives pro-inflammatory polarization and proteostatic stress, whereas hematoma aspiration alleviates these responses and restores reparative functions. Targeting upstream CEBP signaling and enhancing downstream STAT6/LXR pathways may further rebalance microglial phenotypes. Combined surgical hematoma removal with optimized immunoproteasome inhibition thus represents a promising strategy to mitigate neuroinflammation and promote recovery after severe ICH.

## Data Availability

The RNA sequencing data generated in this study have been deposited in the Gene Expression Omnibus (GEO) database under accession number GSE313150. All other data supporting the findings of this study are available from the corresponding author upon reasonable request due to file size limitations and ongoing use in related studies.

## Supplementary Materials

The following supporting information can be downloaded at: https://www.mdpi.com/article/10.3390/cells15080664/s1, Table S1 summarizes the predicted upstream regulators of overlapping differentially expressed genes (DEGs) identified between the I_S_ vs. Normal and I_S-HA_ vs. I_S_ comparisons. Upstream regulator analysis identified key transcription factors (e.g., MYC, YAP1, TP53), cytokines (e.g., TNF, IFNG, IL1B, IL4, IL6), and growth factors (e.g., TGFB1), which were associated with multiple downstream targets, including immunoproteasome subunits Psmb8 (LMP7) and Psmb9 (LMP2), as well as immune- and inflammation-related genes such as ARG1, CD68, CD74, HMOX1, and NFKBIA. Statistical significance (*p*-values) and corresponding target molecules are provided. Figure S1 shows the temporal expression profiles of immunoproteasome subunits following ICH. Western blot analyses of brain tissues collected at 3 h, 6 h, 1 day, 3 days, and 7 days post-ICH demonstrated dynamic changes in proteasome α- and β-subunits, as well as immunoproteasome subunits LMP2 and LMP7. Quantitative analyses normalized to β-actin revealed significant up-regulation of LMP2 and LMP7 at later time points. In addition, Western blot analysis showed that CD68 expression was increased following ICH, with peak levels observed at day 3 post-injury. Immunofluorescence staining further demonstrated colocalization of LMP2 or LMP7 with Iba1^+^ microglia. Figure S2 illustrates the cellular localization of LMP7 after ICH. Immunofluorescence staining showed colocalization of LMP7 with neuronal (NeuN), microglial (Iba1), astrocytic (GFAP), and endothelial (RECA) markers, with predominant localization observed in Iba1^+^ microglia. Confocal z-stack and high-magnification images further confirmed intracellular localization of LMP7 within microglia. Figure S3 demonstrates that hemorrhagic severity regulates immunoproteasome activation and correlates with hematoma volume. Representative brain sections at 3 days post-injury showed differential hematoma sizes across experimental groups. Immunofluorescence staining revealed colocalization of LMP7 with Iba1^+^ microglia. Western blot analyses and quantitative data indicated that severe ICH induced the highest levels of LMP2 and LMP7 expression, which positively correlated with hematoma volume. Figure S4 shows that hemorrhagic severity enhances ER stress and inflammatory signaling following ICH. Western blot analyses demonstrated increased expression of MPO, GRP78, CHOP, and phosphorylated NF-κB in severe hemorrhage, whereas hematoma aspiration partially attenuated these responses. Figure S5 demonstrates immunoproteasome-associated microglial polarization after ICH. The proportion of Iba1^+^CD86^+^LMP7^+^ microglia (pro-inflammatory phenotype) was increased in the I_S_ group and partially reduced following hematoma aspiration, whereas the proportion of Iba1^+^Arg1^+^LMP7^+^ microglia (anti-inflammatory phenotype) was decreased in I_S_ and restored in I_S-HA_. Figure S6 further shows that CD163^+^ microglial polarization is associated with immunoproteasome expression. The proportion of Iba1^+^CD163^+^LMP7^+^ microglia was reduced in the I_S_ group but significantly increased following hematoma aspiration. Data are presented as mean ± SEM. Statistical significance is indicated as * *p* < 0.05, ** *p* < 0.01, *** *p* < 0.001 vs. Normal; # *p* < 0.05, ## *p* < 0.01, ### *p* < 0.001 vs. I_C_; and † *p* < 0.05, †† *p* < 0.01 vs. I_S_.

## Abbreviations

The following abbreviations are used in this manuscript:

AB	Autologous blood injection
Akt	Protein kinase B
Arg1	Arginase-1
BBB	Blood-brain barrier
BI	Balloon inflation (compression)
CD11b	Cluster of differentiation 11b
CD14	Cluster of differentiation 14
CD36	Cluster of differentiation 36
CD68	Cluster of differentiation 68
CD74	Cluster of differentiation 74
CD86	Cluster of differentiation 86
CD163	Cluster of differentiation 163
CEBPA	CCAAT/enhancer-binding protein alpha
CEBPB	CCAAT/enhancer-binding protein beta
CFDA-SE	5-(and-6)-Carboxyfluorescein diacetate succinimidyl ester
CHOP	C/EBP homologous protein
cDNA	Complementary DNA
CNS	Central nervous system
DAPI	4′,6-Diamidino-2-phenylindole
DEGs	Differentially expressed genes
DMEM	Dulbecco’s modified Eagle’s medium
ECL	Enhanced chemiluminescence
ER	Endoplasmic reticulum
FBS	Fetal bovine serum
GEO	Gene Expression Omnibus
GFAP	Glial fibrillary acidic protein
GO	Gene Ontology
GRP78	Glucose-Related Protein 78
HMOX1	Heme oxygenase 1
HRP	Horseradish peroxidase
I_C_	Common intracerebral hemorrhage model
ICH	Intracerebral hemorrhage
IFN-γ	Interferon gamma
IL	Interleukin
IPA	Ingenuity Pathway Analysis
I_S_	Severe intracerebral hemorrhage model
I_S-HA_	Severe intracerebral hemorrhage with hematoma aspiration
IUCAC	Institutional Animal Care and Use Committee
Iba1	Ionized calcium-binding adaptor molecule 1
KEGG	Kyoto Encyclopedia of Genes and Genomes
LMP2	Low-molecular-mass polypeptide 2 (PSMB9; β1i)
LMP7	Low-molecular-mass polypeptide 7 (PSMB8; β5i)
LXR	Liver X receptor
MECL-1	Multicatalytic endopeptidase complex-like 1 (PSMB10; β2i)
mNSS	Modified Neurological Severity Score
MPO	Myeloperoxidase
MYC	MYC proto-oncogene, bHLH transcription factor
NC	Normal control
NeuN	Neuronal nuclei
NF-κB	Nuclear factor kappa B
NFKBIA	NF-κB inhibitor alpha
NIH	National Institutes of Health
O.C.T.	Optimal cutting temperature compound
P1-P2	Postnatal day 1-2
PFA	Paraformaldehyde
PI3K	Phosphoinositide 3-kinase
PSMB8	Proteasome subunit beta type-8
PSMB9	Proteasome subunit beta type-9
PVDF	Polyvinylidene difluoride
qPCR	Quantitative polymerase chain reaction
RBC	Red blood cell
RECA	Rat endothelial cell antigen
RIN	RNA integrity number
RNA-seq	RNA sequencing
SD	Sprague Dawley
SEM	Standard error of the mean
STAT3	Signal transducer and activator of transcription 3
STAT6	Signal transducer and activator of transcription 6
STRING	Search Tool for the Retrieval of Interacting Genes/Proteins
TBST	Tris-buffered saline with Tween 20
TGF-β1	Transforming growth factor beta 1
TNF-α	Tumor necrosis factor alpha
TP53	Tumor protein p53
TPM	Transcripts per million
UPR	Unfolded protein response
UPS	Ubiquitin-proteasome system
YAP1	Yes-associated protein 1
