# Supplementary material for: Immunoproteasome Inhibition Modulates Microglial Polarization to Facilitate Anti-Inflammatory Responses and Hematoma Resolution After Intracerebral Hemorrhage
Source: Cells. 2026 Apr 9;15(8):664. doi: 10.3390/cells15080664 (PMC13115069; doi:10.3390/cells15080664)
Supplement: Supplementary file 1 [file cells-15-00664-s001.zip › cells-4144524-supplementary.pdf]

Supplement figure legend

Table S1. Upstream Regulators of IS-HA vs. IS vs. Normal cross-intersections

Upstream Regulator	Molecule Type	*P*-value	Target Molecules in Dataset
**MYC**	Transcription regulator	1.55E-49	ACTB,ADM,AK2,ANGPTL4,ANXA4,ANXA5,APOC1,ARG1,ARHGAP22,BAG1,BAX,BCL2A1,BIRC5,CASP8,CCND1,CDK1,CDKN1A,CDKN1C,CDKN3,CEBPD,CLEC3B,COL15A1,COL4A1,COL4A2,CRIP1,CSTB,CTSB,CTSD,Ctsl,CXCR4,DCTPP1,DHFR,DNPH1,DUSP2,EBI3,ECM1,EIF4EBP1,EIF5A,FABP5,FBL,FBLN5,FCGR1A,Fcgr3,Fcgr4,FRZB,FTH1,G0S2,GABARAP,GADD45B,GADD45G,GAR1,HMGA1,HMOX1,HP,HSPB1,ID1,IER3,IER5,IFI27,IFI35,IFIT3,IRAG2,IRF7,LDHA,let-7,LGALS1,LGMN,Lyz1/Lyz2,MAP4K1,MCM5,MGP,MRC1,Mt1,MT2A,MYD88,MYL9,NFKBIA,NME2,NQO1,NR1H3,OASL,PGLS,PLAC8,**PSMB8**,RARA,RBP1,RHOC,RIN3,RPIA,RPL10A,RPL11,RPL13,RPL13A,RPL14,RPL18,RPL18A,RPL23,RPL27,RPL28,RPL35,RPL36A,RPL36AL,RPL37A,RPL38,RPL8,RPLP0,RPLP1,RPLP2,RPS12,RPS15,RPS16,RPS19,RPS2,RPS20,RPS21,RPS23,RPS27,RPS27A,RPS28,RPS29,RPS3,RPS5,RPS7,RPS8,RPS9,RPSA,S100A10,S100A6,SERPINH1,SLC11A1,SLC1A5,SNAI1,SNRPD2,SPP1,SQOR,TAGLN2,TALDO1,TGFB1,THBS1,TIMP1,TKT,TMSB4X,TSPO,TXN,UBE2C,UPP1,USP18,WDR4,YBX3,ZFP36
**YAP1**	Transcription regulator	5.98E-43	ADAM8,ALDH1A2,ANGPTL4,ARG1,BAX,BIRC5,BRI3,BST2,CASP8,CCN1,CCND1,CCNF,CD74,CDK1,CDKN1A,CDKN1C,COL4A1,COL4A2,COL6A1,CTSD,CXCR4,CYTIP,DTYMK,EHD1,F13A1,FTL,GINS1,GLRX,H2AX,HMOX1,HP,ID1,IFI27,IFI35,IFNGR1,LBR,LDHA,let-7,LGALS3,LMNA,MRC1,MYL9,NFKBIA,NPC2,NUAK2,**PSMB8**,**PSMB9**,RAB5IF,RNASEH2C,RPL10A,RPL11,RPL13,RPL13A,RPL14,RPL18,RPL18A,RPL23,RPL27,RPL28,RPL35,RPL36A,RPL36AL,RPL37A,RPL38,RPL8,RPLP0,RPLP1,RPLP2,RPS12,RPS15,RPS16,RPS19,RPS2,RPS20,RPS21,RPS23,RPS27,RPS27A,RPS28,RPS29,RPS3,RPS5,RPS7,RPS8,RPS9,RPSA,SLC1A5,SYK,TBC1D2,TGFB1,TGFB1I1,THBS1,TIMP1,TNIP2,TRADD,TRAF2,TXN,WTIP
**TNF**	Cytokine	2.04E-41	ACADS,ACTB,ADAM8,ADM,AK2,ALOX5AP,ANGPTL4,ANPEP,ANXA11,APBA3,APOE,ARG1,ARHGAP22,ARHGDIB,BAX,BCL2A1,BCL3,BIRC5,BST2,BTG2,CASP8,CAVIN3,CCL17,Ccl3,CCN1,CCND1,CD74,CDKN1A,CEBPB,CEBPD,CFD,CH25H,COL15A1,COL4A1,COL4A2,COTL1,CTSB,CTSC,CTSD,CTSZ,CXCR4,CYBA,CYTIP,DUSP2,EBI3,EHD1,EIF4EBP1,ENG,FABP5,FBL,FCER1G,FRZB,FTH1,G0S2,GADD45B,GADD45G,GNA15,GPX1,HLA-DMB,HMOX1,HOPX,HP,HSPG2,ICAM2,ID1,IER2,IER3,IFI27,IFI30,IFI35,IFIT3,IFNGR1,IGFBP2,IGFBP3,IGFBP6,IKBKE,IL17RA,IL18,IL18BP,IL3RA,IL4R,IRF7,LCP2,LDHA,let-7,LGALS3,LITAF,LOXL1,LRG1,LTB,LTBR,LY96,Lyz1/Lyz2,MAP2K3,METRNL,MGMT,MGP,MGST1,MGST2,MOB3C,Mt1,MT2A,MYD88,MYL6,NCF1,NFKBIA,NINJ1,NPM3,NQO1,NR1H3,NRROS,NUAK2,OASL,P2RY2,P2RY6,PDPN,PGF,Pilrb1/Pilrb2,PKMYT1,PLIN2,PLK3,POMC,**PSMB8**,**PSMB9**,PYCARD,RAC2,RARA,RARRES2,RBP1,RBPMS,RCN3,RELB,RFTN1,RIPK3,RPLP0,RPS27A,RPS3,RPSA,S100A10,S100A16,S100A8,S100A9,SDC1,SDC4,SEC61A1,SERPINF1,SLAMF8,SLC11A1,SLC39A8,Slfn2,SMPDL3A,SNAI1,SOCS3,SPP1,SYK,TALDO1,TAP1,TBXAS1,TGFB1,TGFB1I1,TGIF1,THBD,THBS1,TICAM1,TIMP1,TINAGL1,TNFAIP8,TNFRSF1A,TNFRSF1B,TNFSF12,TRADD,TRAF2,TXN,VSIR,XAF1,XDH,ZC3H12A,ZFP36,ZYX
**TGFB1**	Growth factor	1.88E-37	ADM,AHNAK,AIM2,ALOX5AP,ANGPTL4,ANPEP,ANXA11,ANXA2,APOE,ARG1,ARHGAP22,ARHGDIA,ASGR2,AURKA,BAX,BCL3,BIRC5,BMP1,BMP7,BTG2,C1QA,C1QB,CASP8,Ccl3,CCN1,CCND1,CCNF,CD300A,CD68,CD74,CDK1,CDKN1A,CDKN1C,CDKN3,CDT1,CEBPB,CEBPD,CFD,CNN2,COL4A1,COL4A2,COL6A1,COL6A2,COTL1,CTSB,CTSC,CTSD,CTSH,CXCR4,CYBA,DES,DOK1,DUSP2,ECE1,ECM1,EHD1,EIF4EBP1,ENG,ESAM,F13A1,FABP5,FBLN5,FCER1G,FCGR1A,FTH1,FTL,FXYD5,GABARAP,GADD45B,GAL,H2AX,HCAR2,HEBP1,HLA-DMA,HMGA1,HMOX1,HOPX,HSPB1,HSPG2,ICAM2,ID1,IER2,IER3,IFI27,IFI30,IFIT3,IGFBP2,IGFBP3,IGFBP6,IKZF4,IL18,IL4R,KDELR3,LBR,LDHA,let-7,LGALS3,LITAF,LOXL1,LSM2,LSM5,MAP2K3,METRNL,MGMT,MGP,MKNK2,MRC1,MRPS6,MTHFD2,MYD88,MYL6,NCF1,NFKBIA,NR1H3,P2RY6,PDLIM1,PDLIM4,PDPN,PFDN1,POMC,PTH

Supplementary Table S1. Predicted upstream regulators of overlapping DEGs.

Upstream regulator analysis was performed on the overlapping DEGs identified between the

IS vs. Normal and IS-HA vs. IS groups. Key predicted regulators included transcription factors (e.g., MYC, YAP1, TP53), cytokines (e.g., TNF, IFNG, IL1B, IL4, IL6), and growth factors (e.g., TGFB1). Each regulator was associated with multiple downstream targets, including immunoproteasome subunits Psmb8 (LMP7) and Psmb9 (LMP2), as well as immune- and inflammation-related genes such as ARG1, CD68, CD74, HMOX1, and NFKBIA. Significance levels (*p*-values) and the list of target molecules in the dataset are provided in the table. These results suggest that multiple transcriptional regulators and cytokine signaling pathways converge on immunoproteasome components and microglial immune functions after ICH.

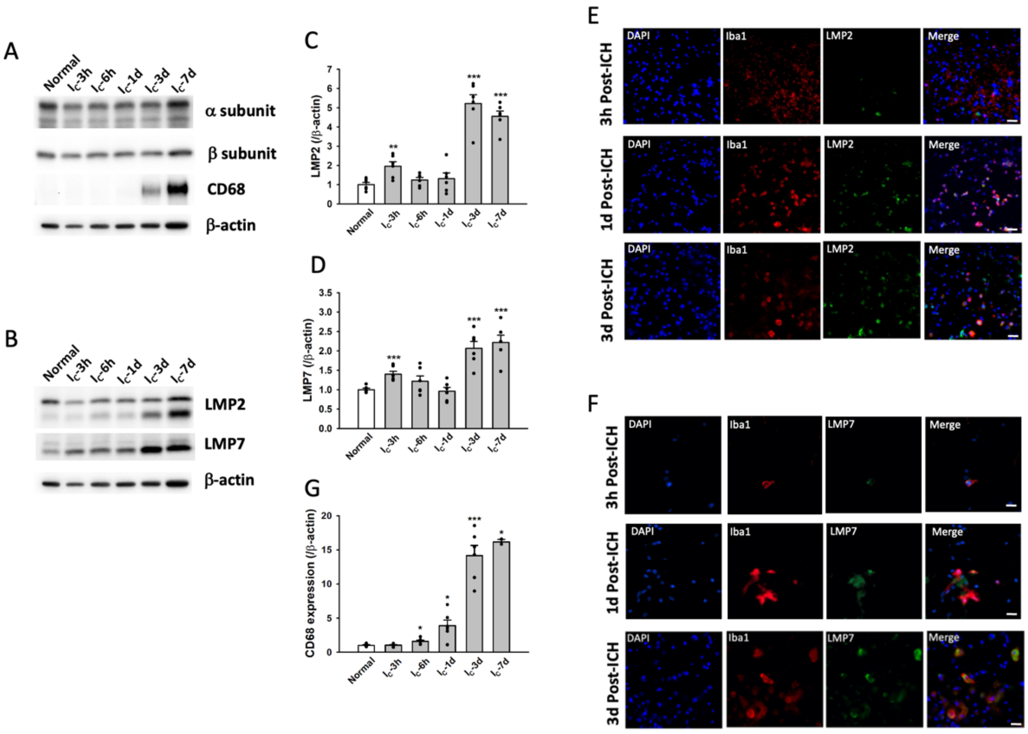

Supplementary Figure S1. Temporal induction of immunoproteasome activation following intracerebral hemorrhage.

(A) Representative Western blot analysis of proteasome α and β subunits and CD68 expression at 3 h, 6 h, 1 day, 3 days, and 7 days post-ICH. (B) Representative Western blot analysis of immunoproteasome subunits LMP2 and LMP7 at the indicated time points. (C–D) Quantification of LMP2 (C) and LMP7 (D) protein levels normalized to β-actin, showing progressive upregulation following ICH, with peak expression at later time points (n = 6 animals per group). (E–F) Immunofluorescence staining showing colocalization of LMP2 (E) or LMP7 (F) (green) with Iba1^{+} microglia (red) at 3 h, 1 day, and 3 days post-ICH. DAPI (blue) marks nuclei. Scale bars = 20 μm. (G) Quantification of CD68 protein levels normalized to β-

actin, showing peak expression at day 3 post-ICH (n = 6 animals per group, except for 7 days where n = 2). Data are presented as mean ± SEM. * $p < 0.05$, ** $p < 0.01$, *** $p < 0.001$ vs. Normal.

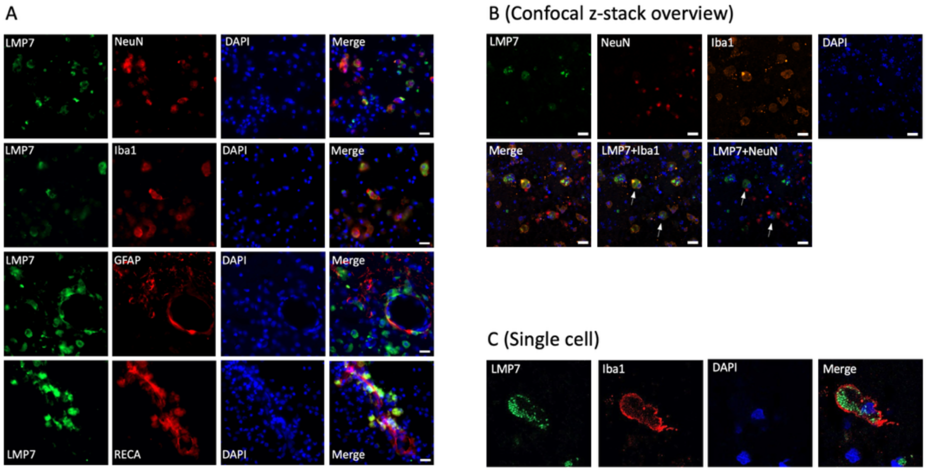

Supplementary Figure S2. Cellular localization of LMP7 in neural cell populations after ICH.

(A) Immunofluorescence staining showing LMP7 (green) colocalization with NeuN⁺ neurons, Iba1⁺ microglia, GFAP⁺ astrocytes, and RECA⁺ endothelial cells; nuclei were counterstained with DAPI (blue). (B) Confocal z-stack images demonstrating spatial overlap of LMP7 with Iba1⁺ microglia but not NeuN⁺ neurons. (C) High-magnification single-cell images confirming intracellular localization of LMP7 within microglia. These findings indicate that immunoproteasome activation predominantly occurs in microglia following ICH. Scale bar = 20 μm.

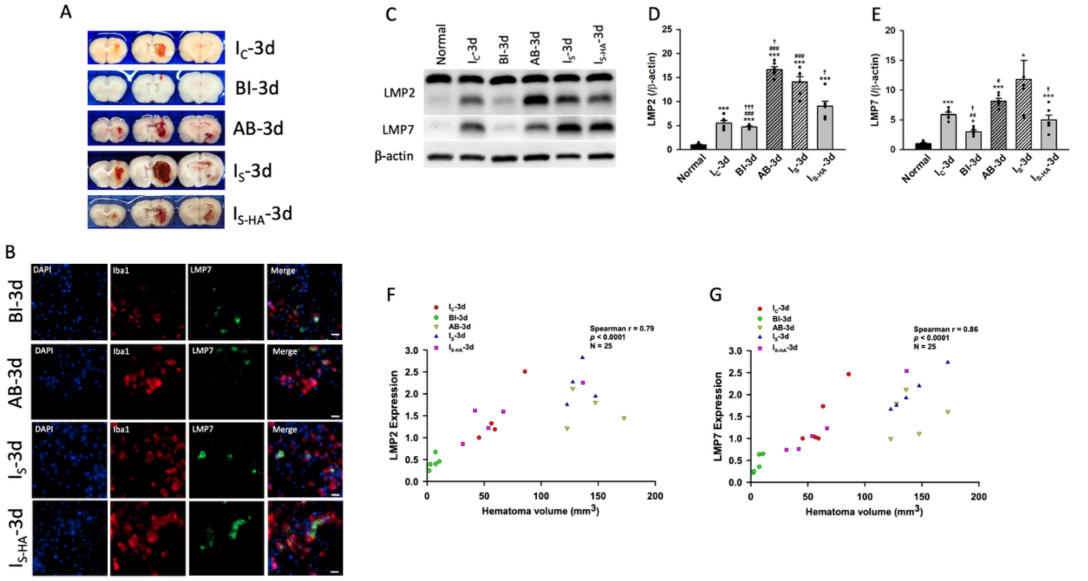

Supplementary Figure S3. Hemorrhagic severity differentially regulates immunoproteasome activation and correlates with hematoma volume.

(A) Representative coronal brain sections at 3 days post-injury illustrating hematoma size in intracerebral injection (I_C), balloon inflation (BI), autologous blood injection (AB), severe ICH (I_S), and hematoma aspiration ($I_{S\text{-}HA}$) groups. BI induced minimal hemorrhage, whereas AB and I_S resulted in progressively larger hematomas, which were reduced following hematoma aspiration. (B) Immunofluorescence staining showing colocalization of LMP7 (green) with Iba1$^+$ microglia (red) in BI, AB, I_S, and $I_{S\text{-}HA}$ groups; nuclei were counterstained with DAPI (blue). LMP7 signals were minimal in BI, moderately increased in AB, and markedly elevated in I_S, with attenuation observed in $I_{S\text{-}HA}$. Scale bars = 20 μm. (C) Representative Western blot analysis of LMP2 and LMP7 expression across experimental groups at 3 days post-injury. (D–E) Representative Western blot analysis and quantification of LMP2 (D) and LMP7 (E) expression normalized to β-actin across hemorrhagic models. Immunoproteasome expression increased in association with hemorrhagic severity, with the highest levels observed in the I_S group (n = 6 animals per group). (F–G) Scatter plots showing the relationship between hematoma volume and LMP2 (F) or LMP7 (G) expression (n = 4 animals per group). Spearman correlation analysis revealed a positive association between hemorrhagic burden and immunoproteasome expression. Data are presented as mean ± SEM. * $p < 0.05$, ** $p < 0.01$, *** $p < 0.001$ vs. Normal; # $p < 0.05$, ## $p < 0.01$, ### $p < 0.001$ vs. I_C; † $p < 0.05$, †† $p < 0.01$ vs. I_S.

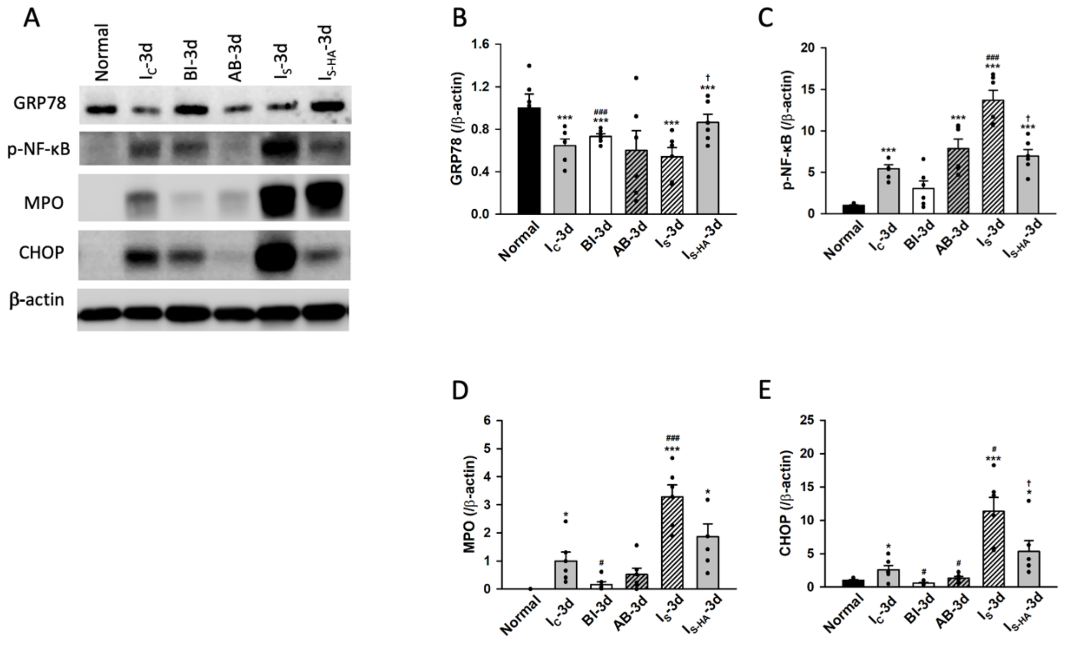

Supplementary Figure S4. Hemorrhagic severity enhances ER stress and inflammatory signaling following intracerebral hemorrhage.

(A) Representative Western blot analysis of GRP78, phosphorylated NF-κB (p-NF-κB), MPO, and CHOP expression across experimental groups (Normal, IC, BI, AB, IS, and IS-HA) at 3 days post-injury. (B–E) Quantification of GRP78 (B), p-NF-κB (C), MPO (D), and CHOP (E) protein levels normalized to β-actin (n = 6 animals per group). Expression of ER stress–related proteins and inflammatory markers was increased in severe hemorrhage and partially reduced following hematoma aspiration. Data are presented as mean ± SEM. * $p < 0.05$, ** $p < 0.01$, *** $p < 0.001$ vs. Normal; # $p < 0.05$, ## $p < 0.01$, ### $p < 0.001$ vs. I_C; † $p < 0.05$, †† $p < 0.01$ vs. I_S.

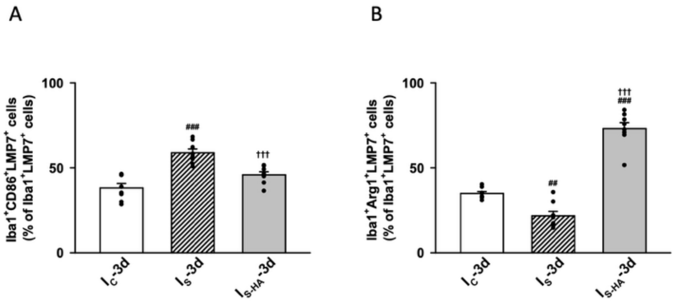

Supplementary Figure S5. Immunoproteasome-associated microglial polarization following intracerebral hemorrhage.

(A) Quantification of Iba1$^+$CD86$^+$LMP7$^+$ cells expressed as a percentage of Iba1$^+$LMP7$^+$ microglia across experimental groups at 3 days post-injury. The proportion of CD86$^+$ (pro-inflammatory) microglia was significantly increased in the I_S group compared with I_C, and partially reduced following hematoma aspiration. (B) Quantification of Iba1$^+$Arg1$^+$LMP7$^+$ cells expressed as a percentage of Iba1$^+$LMP7$^+$ microglia. The proportion of Arg1$^+$ (anti-inflammatory) microglia was decreased in the I_S group but markedly increased in the $I_{S\text{-}HA}$ group. Quantification was performed from 2 animals per group, with 4 fields analyzed per animal. Data are presented as mean ± SEM. ## $p < 0.01$, ### $p < 0.001$ vs. I_C; †† $p < 0.01$, ††† $p < 0.001$ vs. I_S.

A

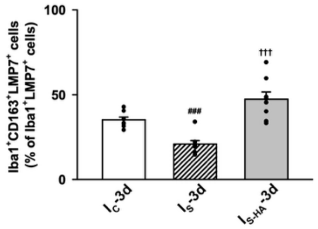

Supplementary Figure S6. Immunoproteasome-associated CD163^{+} microglial polarization following intracerebral hemorrhage.

(A) Quantification of Iba1^{+}CD163^{+}LMP7^{+} cells expressed as a percentage of Iba1^{+}LMP7^{+} microglia across experimental groups at 3 days post-injury. The proportion of CD163^{+} (anti-inflammatory and phagocytic-associated) microglia was significantly reduced in the I_S group compared with I_C, whereas hematoma aspiration markedly increased CD163^{+} microglial polarization. Quantification was performed from 2 animals per group, with 4 fields analyzed per animal. Data are presented as mean ± SEM. ### $p < 0.001$ vs. I_C; ††† $p < 0.001$ vs. I_S.
